# 

**Published:** 1895-09

**Authors:** 


### CONTENTS—SEPTEMBER, 1895.—VOL. XI., NO. 3.

ORIGINAL COMMUNICATIONS—

Hæmorrhagic Malarial Fever. Isaac J. Jones, M. D., Austin . . . . 105
Is the Condition Usually Diagnosed Spider Bite Due to the Bite of a Spider? W. T. Richmond, M. D., Manor . . . . . . . . . . . . . 111

CORRESPONDENCE—

That Resection. Letter from Dr. A. N. Denman . . . . . . . . . . 113

SOCIETY NOTES—

Proceedings Richmond Academy of Medicine and Surgery . . . . . . 118

CURRENT MEDICAL LITERATURE—

Notes on the Recent Progress in Ophthalmology. Frank C. Todd, M. D., Fort Worth . . . . . . . . . . . . . . . . . . . . . . . 121

ABSTRACTS AND SELECTIONS—

Discussion on Erysipelas . . . . . . . . . . . . . . . . . . . . 124
Castration the Remedy for Crime . . . . . . . . . . . . . . . . . 133
The New Woman and the "Bicycle Craze" . . . . . . . . . . . 135
The Indian Hemp Drug Commission . . . . . . . . . . . . . . . . 138
Sufferers Who Long to Die . . . . . . . . . . . . . . . . . . . . 139
The Bicycle from a Surgical Standpoint . . . . . . . . . . . . 140
Bicycling for Women. . . . . . . . . . . . . . . . . . . . . . . 142

EDITORIAL—

The Water Question . . . . . . . . . . . . . . . . . . . . . . . 143
The Only Natural Remedy. . . . . . . . . . . . . . . . . . . . . 148

MEDICAL NEWS AND MISCELLANY—

Numerous Paragraphs of Interest . . . . . . . . . . . . . . . . 150

BOOK NOTICES— . . . . . . . . . . . . . . . . . . . . . . . . . . 154
PUBLISHERS' NOTES . . . . . . . . . . . . . . . . . . . . . . . 155

